# Exploring the REACHOUT Mental Health Support App for Type 1 Diabetes From the Perspectives of Recipients and Providers of Peer Support: Qualitative Study

**DOI:** 10.2196/72779

**Published:** 2026-01-21

**Authors:** Debbie Lam, Poonamdeep Jhajj, Diana Sherifali, Frances S Chen, Tricia S Tang

**Affiliations:** 1 Experimental Medicine Program, Faculty of Medicine, University of British Columbia, Vancouver, BC, Canada; 2 Research Institute, BC Children’s Hospital, Vancouver, BC, Canada; 3 School of Nursing, Faculty of Health Sciences, McMaster University, Hamilton, ON, Canada; 4 Department of Health Research Methods, Evidence, and Impact, McMaster University, Hamilton, ON, Canada; 5 Heather M. Arthur Population Health Research Institute/Hamilton Health Sciences Chair in Inter-Professional Health Research, Hamilton, ON, Canada; 6 Department of Psychology, Faculty of Arts, University of British Columbia, Vancouver, BC, Canada; 7 Division of Endocrinology, Faculty of Medicine, University of British Columbia, 2775 Laurel Street, Vancouver, BC, V5Z 1M9, Canada, 1 604-822-2421

**Keywords:** diabetes, mental health, mHealth, mobile app, mobile health, peer support, qualitative, thematic analysis, type 1, type 1 diabetes

## Abstract

**Background:**

Existing qualitative research in peer support interventions has largely focused on the recipients of support rather than those delivering support. Exploring the perspectives of both roles may provide a holistic understanding of the peer support experience.

**Objective:**

This study elicits the experiences of recipients and providers of support who participated in REACHOUT, a 6-month peer-led mental health support intervention delivered via mobile app for adults with type 1 diabetes. REACHOUT offered multiple support delivery modalities (one-on-one, group-based texting, and virtual face-to-face small group sessions) that could be customized by recipients.

**Methods:**

A total of 32 study participants (recipients and peer supporters) attended focus group discussions following the completion of REACHOUT. Thematic analysis was performed in an inductive approach.

**Results:**

Four major themes were identified by thematic analysis: (1) need for a sense of community and belonging, (2) factors to enhance the recipient-peer supporter experience, (3) key aspects of the peer supporter experience, and (4) importance of personalizing the user experience while using the REACHOUT mobile app. REACHOUT successfully fostered connectedness by bringing together adults with type 1 diabetes who previously felt isolated. Recipients felt greater agency when given the opportunity to self-select a peer supporter. The main factors considered during the matching process included insulin delivery and glucose monitoring systems, duration of diabetes, shared hobbies, life stage, and age. While support was designed to be unidirectional from peer supporter to recipient, the former also derived benefits. Peer supporters expressed the need for greater guidance around navigating boundaries and responding to emotionally charged conversations. Finally, the REACHOUT app was able to accommodate a heterogeneity of support needs by offering one-on-one and group support across multiple communication platforms including text, audio, and video.

**Conclusions:**

The success of peer-led mental health support interventions such as REACHOUT is likely associated with the recipient-peer supporter dynamic. By offering a range of support delivery and communication modalities, participants can better personalize solutions to meet their unique support needs. Understanding the perspectives of both recipients and peer supporters is essential to refining interventions and optimizing digitally delivered mental health support models.

## Introduction

Peer support is a promising self-management strategy to improve emotional health in chronic illness care [1-4]. In the context of diabetes, several systematic reviews of adults with diabetes (both type 1 and type 2 diabetes) have found peer support interventions to be associated with improved clinical, behavioral, and psychosocial (quality of life, perceived social support) outcomes [5-8]. However, to better understand the processes underlying these positive changes, it is important to explore the qualitative experience of giving and receiving peer support.

While qualitative research on peer support interventions has focused largely on the experiences of those who receive support [9-11], there has been a notable increase in studies focused on the individuals who deliver support [12-19]. However, the optimal model for understanding the peer support experience is to explore the perspectives of both parties involved. To date, there have been 4 qualitative studies that have investigated the experiences of both recipients of support and peer supporters in the context of diabetes [20-23]. Of these studies, only 1 recruited adults with type 1 diabetes (T1D) as part of a larger sample [21], while the other investigations targeted adults with type 2 diabetes [20,22,23].

In the era of digital health, peer support models in diabetes have been made more accessible through the shift to virtual platforms such as mobile apps. Such digital peer support programs are especially valuable in rural and remote areas, where access to traditional peer networks and diabetes programs can be limited [24-26]. A systematic review of in-person and technology-mediated peer support for adults with diabetes found that peer support was beneficial in reducing isolation and increasing social support for recipients [27]. However, none of these studies were specific to T1D only. Interestingly, in a review of technology for peer support intervention for adolescents with chronic illness, rather than adults, T1D was the most represented condition [28]. Generally, adolescents with T1D experienced benefits in emotional support and diabetes management [29]. Of the few studies utilizing mobile or web apps for T1D adults, peer support was a secondary feature to self-management behavior education or one of multiple intervention components rather than the main focus [30-33]. As T1D is a lifelong condition, it is important to offer ongoing mental health support to adults living with T1D, especially those facing geographical or resource barriers.

## Methods

### Study Aim

This study aimed to explore the experiences of and perspectives from recipients and providers of support on REACHOUT, a peer-led mental health support intervention for adults with T1D living in rural and remote regions of British Columbia, Canada.

### Study Design

Following the completion of the pilot trial titled REACHOUT, which investigated the feasibility and acceptability of peer-led mental health support intervention delivered by a mobile app, we conducted focus groups with participants of the study. The reporting of methods and findings adheres to the COREQ (Consolidated Criteria for Reporting Qualitative Research) checklist (Checklist 1) [34].

### REACHOUT Intervention Description

Described in detail elsewhere, the REACHOUT pilot investigated the impact of a mobile app that delivered mental health support to adults with T1D living in Interior British Columbia over a period of 6 months. REACHOUT offered multiple support delivery modalities (one-on-one, group-based texting, and virtual face-to-face small group sessions that could be customized by recipients) [35]. Participants include individuals who receive support (recipients) and those who provide support (peer supporters). In this paper, the term “participants” will only be used when addressing both recipients and peer supporters. The eligibility criteria for recipients were as follows: (1) be diagnosed with T1D, (2) be at least 18 years or older, (3) speak English, (4) have access to the internet and/or a smartphone, (5) live in the interior region of British Columbia, and (6) have a mean subscale score of ≥2 on the type 1 Diabetes Distress Scale [36]. Peer supporters had similar requirements with the exceptions of criteria 5 and 6. They also had to be willing to complete a 6-hour training program. Training components and competency evaluation are published elsewhere [37]. It should be noted that if asked a medical question by recipients, peer supporters were instructed to refrain from answering and defer to the diabetes nurse educator.

The REACHOUT app offered multiple support delivery modalities including one-on-one support provided by a recipient-selected peer supporter, group texting support via the 24/7 chat room, and small group face-to-face support via video huddles and happy hours. Recipients were encouraged to use any or all modalities as frequently as desired. Peer supporters were invited to attend virtual wellness sessions to debrief their experiences as well as receive their own emotional support. Finally, the ongoing monitoring of group-based communication exchanges was performed by the research team, and fidelity assessments were conducted at 1, 3, and 5 months of the intervention with all participants.

### Ethical Considerations

This qualitative descriptive study was approved by the University of British Columbia Behavioural Research Ethics Board (H20-00276). Prior to focus groups, participants provided e-informed consent using REDCap (Research Electronic Data Capture) electronic data capture tools hosted at the University of British Columbia [38,39]. To maintain privacy and confidentiality, recordings were anonymized to omit personal identifying information and stored securely. Only the study team could access study data. Upon completion, participants received a CAD $25 (approximately US $18) e-gift card.

### Participant Recruitment and Sampling

Following the completion of the pilot trial REACHOUT, all those in recipient roles were contacted by a research assistant and invited to the postintervention focus groups to share their experience with the REACHOUT program and app and suggestions for improvement. Only peer supporters who had been paired with recipients were invited to join the postintervention focus groups. Those who provided consent were interviewed.

### Data Collection and Analysis

Focus groups were conducted online using Zoom; video and audio were recorded and later transcribed. Led by a female researcher (TST), focus groups were stratified into recipient versus peer supporter-only membership with approximately 6 individuals per group. The interview guide (Multimedia Appendix 1) used open-ended questions and prompts to elicit discussion around their experience in the program, peer support interactions, and app usage. Follow-up questions were posed if clarification or explanation was needed.

Recordings were transcribed verbatim and anonymized with participant roles (recipient or peer supporter) identified to capture perspectives from both groups. Transcripts were analyzed using NVivo V.14 software package [40]. Guided by an interpretivist research paradigm, which centers around subjective experiences [41], we selected an inductive thematic approach to support the possible variation of participant perceptions. Following Braun and Clarke’s 6 phases of thematic analysis, 1 coder (DL) participated in transcribing the data and another coder (PJ) who had no involvement in the interview guide development, interviews, and transcription familiarized themselves with the transcripts [42]. Both coders discussed initial ideas before independently performing open coding. The coders discussed the findings after every round of coding to enhance reflexivity and iteratively refine a unified codebook. Independently coded transcripts were combined, and codes were sorted and combined to form themes and subthemes. Themes and subthemes were reviewed and refined with clear definitions and names. Findings and any discrepancies were discussed with the principal investigator (TST) and another coauthor member (DS) who was not involved in the interview guide creation and interviews. Moreover, this was a recursive process where analysis phases moved back and forth as needed [42].

### Positionality Statement

Our multidisciplinary team comprises cisgender, heterosexual women from East Asian, South Asian, and European settler backgrounds. TST has over 25 years of experience working in peer support, and her research focuses on developing models to improve mental health outcomes in high-risk and medically underserved communities. DS has over 25 years of research working in diabetes self-management at the community and provider level. FSC has over 20 years of experience working on topics related to stress, social support, and social connection and contributes a behavioral science perspective. DL and PJ are early-career researchers with master’s and medical graduate training. All authors are living in urban centers and are cognizant of their own privileges and practice reflexivity to ensure that priorities of the diabetes community are represented throughout the research process.

## Results

### Description of Sample

In total, 32 study participants (17 recipients and 15 peer supporters) who completed the REACHOUT intervention were recruited and interviewed from August to October 2022. The characteristics between focus group participants compared to nonrespondents in the pilot study population are noted in Multimedia Appendix 2. There were 9 focus groups lasting 60‐90 minutes, 4 recipient-only groups, and 5 peer supporter-only groups. As summarized in Table 1, participants were predominantly women and Caucasian, with a mean age of 48 (SD 16.3; range 23‐76) years and an average of 24 (SD 18.1; range 0‐65) years living with diabetes. Most participants received postsecondary education and had a household income greater than CAD $70,000 (approximately US $50,505).

**Table 1.** Interviewed recipients’ and peer supporters’ baseline characteristics.

	Total focus group participants (n=32)	Recipients (n=17)	Peer supporters (n=15)
Age (y), mean (SD)	48 (16.3)	48 (16.6)	50 (16.4)
Diabetes duration (y), mean (SD)	24 (18.1)	25 (18.5)	23 (18.2)
Women, n (%)	26 (81)	15 (88)	11 (73)
Marital status, n (%)			
Never married	9 (28)	6 (35)	3 (20)
Married or living with a partner	20 (63)	10 (59)	10 (67)
Separated or divorced or Widow	3 (9)	1 (6)	2 (13)
Ethnicity, n (%)			
Aboriginal	1 (3)	1 (6)	0 (0)
Aboriginal/Caucasian	1 (3)	1 (6)	0 (0)
East Asian (Chinese, Korean, Japanese)	1 (3)	0 (0)	1 (7)
Caucasian	29 (91)	15 (88)	14 (93)
Education, n (%)			
High school graduate (or equivalent)	3 (9)	3 (18)	0 (0)
Some college or technical school	7 (22)	4 (24)	3 (20)
College graduate	10 (31)	3 (18)	7 (47)
Graduate degree	12 (38)	7 (41)	5 (33)
Pretax household income (CAD $), n (%)			
<70,000 (approximately US $50,505)	10 (31)	7 (41)	3 (20)
>70,000 (approximately US $50,505)	17 (53)	5 (29)	12 (80)
Declined to answer	5 (16)	5 (29)	0 (0)
Employment, n (%)			
Full-time job	12 (38)	6 (35)	6 (40)
Part-time job	6 (19)	5 (29)	1 (7)
Retired	6 (19)	2 (12)	4 (27)
Other	7 (22)	4 (24)	3 (20)
Declined to answer	1 (3)	0 (0)	1 (7)

### Themes

Four overarching themes were identified and related to participants’ experiences in the peer support intervention and on their user experience with the mobile app delivery (Table 2).

**Table 2.** Four major themes were identified by thematic analysis with subthemes that capture similarities and differences within and across recipient and peer supporter groups.

Theme	Recipient	Peer supporter	Both group
Need for a sense of community and belonging	—^a^	—	Giving and receiving unconditional support Reducing isolation in rural communities Learning from real-life experiences of T1D peers
Factors to enhance the recipient-peer supporter experience	Ability to select a peer supporter	—	Modality and frequency of communication
Key aspects of the peer supporter experience	—	Supporting peer supporters in their role Benefits of being a peer supporter Challenges of being a peer supporter	—
Importance of personalizing the user experience while using the REACHOUT mobile app	—	—	Varied preferences in peer support Adapting the mobile app to fit user expectations

a Not applicable.

#### Theme 1: Need for a Sense of Community and Belonging

For recipients and peer supporters, REACHOUT created a safe environment to build and strengthen connections with other adults who shared the lived experience of T1D. This sense of belonging and community spirit manifested in different ways.

##### Subtheme A: Giving and Receiving Unconditional Support

The intervention created a space to express concerns without fear of judgment or rejection. Participants who had felt completely alone in the past finally found their “tribe”—a community that experienced and understood the same fears, frustrations, and emotional burdens of T1D.

*The whole thing has been just so rewarding and I think it’s kind of brought me out a little bit too. Like being able to be who I am and not be judged it’s like – it’s just this community. Being able to kind of hop into the chat and say, “Oh yeah this is what happened to me” or you know, just that common sharing. It’s been huge.*

[Peer supporter 5-2]

Initially, some participants were hesitant to engage in group activities such as face-to-face virtual sessions because the possibility of meeting peers who were managing their diabetes “perfectly” could trigger feelings of inadequacy or resentment. However, once the intervention started, they realized others were willing to be vulnerable. For example, when some participants disclosed perceived self-management failures in the 24/7 chat room, they were met with empathy and validation. After this precedent was established, others felt safe to reveal moments of insecurity and self-blame.

*It was really nice to know when you’re like, “I’m doing everything possible to keep my blood sugar stable right now and for the life of me they’re on the higher side. I don’t know why.” But knowing other people are like, “Yeah, isn’t that frustrating,” like they get it because they live it. It’s not like your [endocrinologist], it’s nice to hear it from somebody who lives it, I don’t feel so alone in the world.*

[Recipient 6-3]

##### Subtheme B: Reducing Isolation in Rural Communities

Coming from rural and small communities across Interior British Columbia, many recipients and peer supporters had never encountered another T1D adult in their local community. This sense of loneliness was particularly pronounced for individuals diagnosed late in life (eg, 45 years and older).

*It seems like we grew up in a smaller town, and there wasn’t anybody that had diabetes that I knew, and then going through the other parts of my life, I didn’t have really anybody to talk to.*

[Recipient 4-2]

Although REACHOUT was a virtual intervention, participants were comforted knowing that peers resided in nearby towns. When browsing through the peer supporter library, participants were able to identify the general location where each peer supporter lived and, therefore, felt reassured that face-to-face support was accessible if needed. As part of the REACHOUT community, participants were not left to cope with the struggles of T1D on their own.

*I thought it was really nice to connect with people, maybe not totally in my community. But certainly, there have been a great number of people within an hour’s drive that’s connected with and there’s just something about that to know that you’re not alone in your little portion of the world.*

[Recipient 6-2]

##### Subtheme C: Learning From Real-Life Experiences of Peers With T1D

With REACHOUT, participants had direct access to the most reliable and high-quality T1D information including “real-world” experiences from adults who used insulin, insulin pumps, and continuous glucose monitors daily. The mobile app offered different mechanisms to obtain the knowledge needed. For instance, in the 24/7 chat room, participants posted updates regarding changes to health insurance coverage or, during the COVID-19 period, shortages in various diabetes supplies. This platform was also a place to pose questions and elicit differing perspectives from both recipients and peer supporters. For example, participants who were considering transitioning to a different insulin pump or continuous glucose monitoring device could hear opinions from peers from diverse lifestyles and backgrounds.

*It was cool to hear firsthand information from somebody’s experience, say about the Omnipod or the Medtronic or Dexcom or whatever. I think that’s invaluable, rather than just going to a doctor or endocrinologist and just a medical professional, which is still really good information but to get the user’s perspective on something is kind of for sure.*

[Peer supporter 7-1]

Notably, how participants preferred to learn varied. Those who were not comfortable posting messages or disclosing personal experiences still enjoyed reading the discussion threads and exchanges in the 24/7 chat room. Many participants routinely checked the app to read the most recent conversation and updates. While not directly participating, participants who passively monitored the exchange of dialogue derived substantial benefits.

*In my journey over the years with diabetes, I just felt so alone, so this app has been – just knowing it’s there has been huge. I’m kind of a classic introvert – I don’t really go on and participate actively on it, but I do on in and I read the conversations and just I love it. Please don’t underestimate power of that because it’s really been a big thing for me.*

[Peer supporter 7-2]

### Theme 2: Factors to Enhance the Recipient-Peer Supporter Experience

Factors related to one’s experience with REACHOUT were largely dependent on the quality of the recipient-peer supporter relationship. Many found their peer supporter extremely helpful and valued their time, but the strength of their relationship was influenced by various contributing factors.

#### Subtheme A: Ability to Select a Peer Supporter

Recipients felt empowered by the opportunity to choose their peer supporter. Some sought identical counterparts, while others envisioned their peers as potential mentors. The criteria that each recipient used to choose their peer supporter were unique and personal. The main factors included diabetes management system, duration of diabetes, shared activities, life stage, and age.

According to some recipients, diabetes and management-related factors weighed heavily into the selection process. For example, some recipients were seeking a peer supporter who had been living with diabetes for as long, if not longer, than themselves. Others felt a greater kinship with peer supporters using the same continuous glucose monitoring or insulin pump.

*I looked at not necessarily insulin type, but just device that they might be using. And for me, the Dexcom was new so I wanted somebody who knew and used the Dexcom. So that was some of my criteria when I started to go through the list. I don’t need to read the other fifteen that don’t use a Dexcom, that was a clear priority for me.*

[Recipient 6-3]

Lifestyle factors also factored in prominently when selecting a peer supporter. For instance, recipients who enjoyed exercising or engaging in outdoor sports preferred an equally active peer supporter. Having shared hobbies enhanced the quality of recipient-peer supporter relationships and extended conversations beyond the boundaries of diabetes. In contrast, in the absence of similar interests, some recipients found it difficult to establish meaningful and sustained rapport with their peer supporters.

*Device for me wasn’t as important. Cause I’ve been on both injections and pump. So for me, mostly activities and hobbies. And someone that liked to travel as well, cause I always find that quite daunting but I want to do more of that so yeah. I found a good person for that*.

[Recipient 8-4]

The stage of life was equally important. For example, young mothers gravitated toward selecting peer supporters who were also raising children. As expected, navigating both diabetes and parenthood created strong connections. Similarly, older recipients who were retired understood the priorities and pace of others who were also no longer in the workforce.

*I picked someone who was in a similar life stage as me, cause I’ve had diabetes for 30 years I don’t really need advice on how to treat my diabetes. For me, it was much more the mental health connection and then transition to this new part of my life of being a mom. Because stuff would come up and I’d be like oh, my gosh, how do you deal with this? How do you prioritize a crying baby verses a low? So that for me was great.*

[Recipient 9-1 ]

Age and/or length of diabetes experience emerged as critical factors in the selection process. Some recipients intentionally chose older peer supporters who had a lifelong journey with diabetes as they envisioned having a mentor who could provide insight on what challenges to expect over time. Rarely did recipients choose peer supporters who were much younger than themselves.

*Someone [who] was male, and older than me. So I can relate to what they’re going through, and someone who has had diabetes for longer than I have. So it’s quite focused of what I was looking for. I was able to be paired up with someone who was in my position, but a couple years down the road.*

[Recipient 8-3]

#### Subtheme B: Modality and Frequency of Communication

Video conferencing was the most preferred modality, as it allowed for the 2 parties to observe facial expressions and body language. Different communication methods were utilized for different functions. Direct messaging, texting, and emails were ideal for quick communication such as check-ins and meeting coordination. If both parties were amenable to investing greater effort and commitment, more substantial conversations took place through video conferencing or phone calls.

Consistency formed the foundation of a strong recipient-peer supporter relationship. Initially, weekly communication was needed to establish and build rapport. However, as the relationship matured, for some, the frequency of contact slowed down as people had other competing life demands such as full-time jobs or home responsibilities. Mid-intervention, many acknowledged that the ideal schedule was contact once every 2 weeks.

*I liked that it was once a week in the beginning. I think it gave you a lot of opportunity to get to know each other, tell each other your diagnosis story and then from there on. I think I did realize with my peer supporter when we started, when we were meeting every week that we almost were running out of things to update each other on or talk about. And then every two weeks was really great and then we had some things to share over the last two weeks.*

[Recipient 8-1]

### Theme 3: Key Aspects of the Peer Supporter Experience

The cornerstone of a peer-led intervention is the peer supporters who deliver mental health support. Although the goal of REACHOUT was to provide support to recipients, the sustained quality of the 6-month intervention provided opportunities for peer supporters to be nurtured as well.

#### Subtheme A: Supporting Peer Supporters in Their Role

To function effectively in their role, peer supporters underwent a 6-hour training. According to peer supporters, the most instrumental training activity was “role-plays.” Not only did role-plays allow trainees to practice newly developed skills, but these simulated scenarios helped build their self-confidence and preparedness.

During the intervention, peer supporters appreciated having a workbook with structured activities to lead their recipients through. These activities served as a valuable foundation for conversations that would not occur organically—for example, identifying personal values and exploring sources of diabetes distress.

Furthermore, peer supporters benefited from attending wellness sessions hosted by the research team. Wellness sessions were Zoom-based and provided the opportunity for peer supporters to share stories, voice concerns, and pose questions to one another. Moreover, these discussions fostered camaraderie among peer supporters while navigating inherent challenges in their support roles.

*I think every [Wellness] session – I found important, because there’s always something new that you can take away. And then, if there’s a question that I have, [I] can actually ask during those sessions. “Okay, you know. Great. I’m on the right track,” you know as well and then, “I’m following what I supposed to be following and doing what I’m supposed to be doing with the peers.”*

[Peer supporter 1-1]

#### Subtheme B: Benefits of Being a Peer Supporter

Peer supporters derived deep satisfaction and intrinsic reward from their role, finding genuine fulfillment from providing mental health support to other adults with T1D. Through acts of altruism and compassion for the T1D community, they experienced satisfaction knowing that their contribution added meaning and value to the lives of their recipient.

Many peer supporters realized that their relationship with their matched recipient was mutually beneficial. Not only did peer supporters deliver emotional support, but recipients also shared their knowledge, coping strategies, and perspectives. Additionally, many peer supporters discovered a renewed connection with their own diabetes journey and engaged in self-reflection and self-development.

*[My recipient] was fairly newly diagnosed, within the last year, and it’s been 11 years for me. I benefited a lot from talking with her. It kind of re-engaged me in diabetes. I think I realized I*’*ve been coasting, and I needed to kind of re-engage, and I think that was really important for me.*

[Peer supporter 2-2]

#### Subtheme C: Challenges of Being a Peer Supporter

Not all peer supporters had recipients who reciprocated with the same level of enthusiasm. Rather than feeling rejected if their recipient did not respond immediately, some peer supporters did not take it personally. Moreover, peer supporters found it challenging to sustain consistent communication with their recipients, especially in the last half of the intervention. Peer supporters tried to understand their recipient’s perspectives by acknowledging the demands of personal and professional lives.

*I found sending a text- something, I felt like I was chasing her. And I would think, “Oh maybe she doesn’t want to talk to me anymore,” “Maybe she’s had enough,” or, “Maybe I’m doing something wrong,” but it wasn’t anything like that at all. It was just she was busy; she has a job and family.*

[Peer supporter 5-3]

Some peer supporters struggled to deepen their conversations when recipients appeared to be reluctant to broach more sensitive topics. At times, peer supporters adhered to surface-level conversations so as to not “over-step.” As such, peer supporters suggested having more guidance on how to navigate boundaries and tips for gauging the depth recipients seek from relationships.

*I didn’t bring up the underlying issues as much as I would have expected, perhaps because I wasn’t quite versed in how to bring those up. I didn’t know if it was appropriate for me to kind of prod a little bit. [… ] I felt a little bit at a loss of how to bring up like these big concepts, psychological issues and things like that. There was definitely stuff going on, but it was hard for me to get them to speak about some of those things*.

[Peer supporter 2-1]

Conversely, some peer supporters encountered recipients who openly shared their feelings and concerns, which posed a different challenge as it triggered feelings of worry and inadequacy. Peer supporters were seeking greater instructions on how to navigate these emotionally charged conversations. Two potential solutions suggested were (1) establishing clear guidelines on how to respond to questions requiring escalation to a health professional and (2) providing a set of prepared questions to ask when these situations arose.

*I’m not gonna lie, I was a little bit stressed if this person was really in distress, because I don’t know if I was like, “Jeez, like I don*’*t know if I can be the guy that’s going to help this person.” But I was pretty fortunate, [my recipient] just wants someone to talk to, basically, which worked out well for me.*

[Peer supporter 7-1]

### Theme 4: Importance of Personalizing the User Experience While Using the REACHOUT Mobile App

Participants (recipients and peer supporters) had four ways to engage with others on the REACHOUT mobile app: (1) direct messaging, (2) 24/7 chat room, (3) virtual happy hours, and (4) virtual huddles.

#### Subtheme A: Varied Preferences in Peer Support

The 24/7 group chat room served as a central feature of the app with a significant amount of activity. Most participants referred to the 24/7 chat room to pose questions, share stories and updates, and initiate discussions. The high level of engagement led many participants to habitually check the chat to stay informed. For some, monitoring the 24/7 chat room was a part of their daily routine, as participants could obtain new information as well as be exposed to a diverse range of topics.

Alternatively, some found the continuous flow of information in the 24/7 chat room to be overwhelming. Specifically, it was burdensome to sift through a high number of messages to find discussions of personal relevance. For example, while the majority of participants discussed insulin pumps, it alienated the few individuals who used multiple daily injections. In extreme cases, some participants deactivated the notifications setting for the 24/7 chat room.

*Like it was overwhelming right at the beginning [from the 24/7 chat room], and so I turned off the notifications but then I got it out of the habit of checking, so I missed a whole bunch of stuff, me and my mentor were communicating through text, so I didn’t really have to worry about going back into the app.*

[Recipient 9-1]

Virtual huddles and virtual happy hours were 2 additional support delivery mechanisms offered. The former was a larger-group interactive webinar led by peer supporters and/or professionals and required fewer social demands or active participation. The latter involved a smaller, intimate group discussion led by a peer supporter and fostered open and relaxed conversations beyond their one-on-one peer support relationship. These 2 support modalities cater to diverse personality types and needs.

#### Subtheme B: Adapting the Mobile App to Fit User Expectations

App usability issues centered largely around the lack of logical structure and flow of exchanges within the 24/7 chat room. Because participants had the option of responding within a thread or creating a new thread, conversations often seemed disjointed. As a result, many suggested creating more topic-focused discussion boards as “exit ramps” from the 24/7 chat room, allowing participants to select personally relevant information in a structured way. Participants also suggested a keyword search feature. This element would streamline the process of finding specific information without the need to scroll through recent posts. To increase accessibility for people with different reading abilities, participants suggested that the app be available on bigger devices such as tablets or computers.

Finally, the mobile app experienced various bugs. For research purposes, this app was launched on a testing platform that required participants to log in with their credentials every 3 months. This issue led to widespread frustration and confusion among participants who lost access unexpectedly. Additionally, there were bugs in the video feature, which made it difficult for participants to connect unless they used platforms outside of the mobile app (eg, Zoom, Facetime). Future improvements to fix these bugs would ensure a smoother and more reliable user experience.

*I guess I went to log on the other day I wasn’t sure when it ended, and I was quite sad when I didn’t have access anymore, to go on and read the stuff I was used to reading each day so that was kind of, that was nice. Well, it wasn’t nice that I couldn’t get on but it was nice, yeah.*

[Recipient 4-2]

## Discussion

### Principal Findings

This study explored recipients’ and peer supporters’ experiences with and perspectives on REACHOUT, a peer-led mental health support intervention for adults with T1D living in rural and remote regions of British Columbia. Our results identified four major themes: (1) Need for a sense of community and belonging, (2) Factors to enhance the recipient-peer supporter experience, (3) Key aspects of the peer supporter experience, and (4) Importance of personalizing the user experience while using the REACHOUT mobile app.

### Comparison to Prior Work

Consistent with our findings, the need for community and belonging, especially for geographically marginalized individuals, has also been reported in the literature. For instance, a systematic review of 12 qualitative studies on health care access for rural patients with chronic diseases found that a sense of group connection in rural areas mitigates feelings of vulnerability [43]. Similarly, Joensen et al [44] noted that while a feeling of inclusion contributes to health promotion, it is often lacking in daily life for individuals with T1D. Thus, a mobile app such as REACHOUT is especially valuable in addressing these gaps in remote and underserved communities.

With REACHOUT, recipients had the agency to choose a peer supporter based on personally relevant factors. This choice-based model deserves consideration, as it may optimize the recipient-peer supporter match [12,35]. Our data also suggest that successful pairs often referred to one another as “friends,” which supports the idea that effective emotional support is built upon friendship and trust [17,22]. To enhance participant satisfaction, future peer support studies should adopt this recipient-driven matching process as recipients are in the best position to understand their own unique support needs.

While the one-on-one and group support delivery mechanisms address different support needs, many recipients expressed greater value for the former. The advantages of personalized individual relationships address the limitations inherent in group settings. For example, in an intervention of peer support meetings for adults with T1D focusing on insulin pumps, dissatisfied participants reported a lack of relevance in the discussion topics, hindering their ability to speak about topics that mattered to them [45]. Incorporating modalities that allow recipients to seek both group-based and one-on-one peer support within the same intervention promotes greater support customization for each user. Subsequent mental health support models should prioritize flexible delivery options that balance individualized support with opportunities for group engagement.

As participation in group activities within the mobile app was optional, we observed varying levels of engagement. Passive participation, characterized by viewing (vs posting) 24/7 chat room exchanges, was the most common. Participants engaged in “lurking” behavior, which involved routinely checking the chat room, gleaning value in reading anecdotes and being exposed to new topics related to T1D. “Lurking” was also observed in an online community–based peer support forum for in-hospital patients. This study found that 7 of 30 participants opted not to post yet still experienced a positive impact on emotional well-being [46]. Additionally, Tang et al [47] found that adults with T1D who passively engaged with the digital support platform (ie, ‘lurkers’) reported greater reductions in stigma-related distress compared to active posters. These findings highlight the role of passive engagement in mental health interventions as a strategy for mitigating “social risks” [47,48]. An in-depth examination of the mental health benefits associated with passive participation on digital platforms is warranted.

While not anticipated by peer supporters, the flow of support with recipients was bidirectional. However, the content of the “give and take” exchange likely encompassed a range of topics not necessarily diabetes-specific. Nonetheless, this opportunity for mutual sharing was also cited in a systematic review of qualitative peer support studies for chronic diseases [49]. Recognizing this reciprocity as an unintentional intervention, peer support studies should routinely assess changes in outcomes for both recipients and peer supporters. Clearly, peer support fosters emotional well-being for both parties.

Ensuring ongoing support for peer supporters beyond the initial training phase is essential for peer supporter effectiveness and well-being. Our intervention addressed this need by offering peer wellness sessions, a space for peer supporters to share successes and challenges. Not surprisingly, emotional investment leading to exhaustion can harm the mental health of peer supporters [50]. Thus, having an environment to express frustrations in real time such as how to deal with nonresponsive recipients or navigate emotionally charged conversations could potentially prevent burnout or dissatisfaction. Therefore, implementing regular communication or check-ins could enhance peer supporters’ experience and overall intervention effectiveness.

### Limitations

First, this study only recruited matched peer supporters (vs unmatched peer supporters). Perspectives from unmatched peer supporters were not captured. Future studies should consider interviewing those peer supporters who did not participate in the one-on-one support component but had access to other support delivery features. Second, this sample was self-selected and possibly more engaged and enthusiastic than other participants. This may limit the representativeness of the original REACHOUT cohort. While we compared the characteristics of the consenting and nonconsenting sample, future studies should ensure representation across different levels of engagement. Third, the socioeconomic background for participants was relatively high. Because we did not overrecruit for individuals with lower levels of income or education, the diversity of experiences captured may be skewed. Finally, the study targeted the rural and remote communities of Interior British Columbia; therefore, the results may not be generalizable to other geographically marginalized populations in BC or Canada.

### Conclusions

Peer support is increasingly recognized as a critical component for mental health interventions in T1D. While research has focused largely on recipients of support, our study also considered perspectives of individuals delivering support, providing a holistic view. More importantly, it is the recipient-peer supporter dynamic that most likely drives the success of the implementation of the REACHOUT program and impacts mental health outcomes. Only by understanding the experiences of both parties can we refine our interventions to provide the optimal mental health support model.

## Supplementary material

10.2196/72779 Multimedia Appendix 1 Interview guide created by the principal investigator and research team to guide focus group discussions.

10.2196/72779 Multimedia Appendix 2 Interviewed participants’ baseline characteristics compared to nonrespondents from the larger pilot participant population. Mann-Whitney *U* tests were applied for continuous variables and the Fisher exact test for categorical variables.

10.2196/72779 Checklist 1 COREQ: 32-item checklist.

## Abbreviations

COREQ: Consolidated Criteria for Reporting Qualitative Research
REDCap: Research Electronic Data Capture
T1D: type 1 diabetes
