# Supplementary material for: Exploring the REACHOUT Mental Health Support App for Type 1 Diabetes From the Perspectives of Recipients and Providers of Peer Support: Qualitative Study
Source: JMIR Diabetes. 2026 Jan 21;11:e72779. doi: 10.2196/72779 (PMC12822868; doi:10.2196/72779)
Supplement: Multimedia Appendix 1 [file diabetes-v11-e72779-s001.docx]

**Multimedia Appendix 1.**

Interview guide created by the principal investigator and research team to guide focus group discussions.

| Participant Only Questions | | Peer Supporter Only Questions | |
| --- | --- | --- | --- |
| Main question | **Additional guiding questions** | **Main question** | **Additional guiding questions** |
| How was it being a participant in REACHOUT? | - What were the positive aspects, if any, of being a participant? - What were the negative aspects, if any, of being a participant? - What would you tell people who are interested in becoming in participating in REACHOUT in the future? | How was it being a Peer Supporter? | - What were the positive aspects, if any, of being a Peer Supporter? - What were the negative aspects, if any, of being participant? - What would you tell people who are interested in becoming a Peer Supporter in the future? |
| What was your experience with the one-on-one support delivered by your Peer Supporter? | - How useful was the Peer Supporter Profile library? - How good a match were you and your Peer Supporter? - What, if anything, would you change about the matching process? - How often, if at all, do you think participants should switch to a different Peer Supporter? | How useful, if at all, was the Peer Supporter Training in preparing you for your role as a Peer Supporter? | - What skills did you use? - What skills did you not use? - For the next training program, what skills and/or content should we keep? - What skills/content should we take out? - What skills and/or content should we add? |
|  | | As a Peer Supporter, what additional support, if any, do you wish you had during the REACHOUT program | - How useful were the Peer Supporter Wellness sessions? - How often should we conduct Peer Supporter Wellness sessions? - What topics would you like to discuss in the Wellness sessions? |
|  |  | What was your experience delivering one-on-one support to your participant? | - How useful was the Peer Supporter Profile library? - How good a match were you and your participant? - What, if anything, would you change about the matching process? - How often, if at all, do you think participants should switch to a different Peer Supporter? |
| What was your experience using the REACHOUT App? | | - What features on the App did you use the most? - What features on the App did you use the least? - If you had a wish list, what modifications or additions would you make to the App? - How well do you think the research team prepared you to use the App? - What do we need to include in an orientation to the App the next time we conduct REACHOUT? | |
| What was your experience with the different support delivery features of the App? | | - What was your experience with the 24/7 chat room? - What was your experience with the Video Huddles? - What was your experience with the discussion boards? - What was your experience with the Virtual Happy Hours? | |
| What, if anything, would you change about the intervention itself? | | - What is the ideal frequency of contact? - What methods of contact do you use most frequently to communicate with your participant? - What other support features should we add? Or take away? | |
| How well do you feel the research team supported you? | | - How satisfied were you with your assigned research assistant? - What did you think about the 1-, 3-, and 5- months fidelity phone calls? - What are the ideal number of treatment fidelity calls should the research team? What interval should this be? - What changes would you make for the future? | |
