# Supplementary material for: Exploring the REACHOUT Mental Health Support App for Type 1 Diabetes From the Perspectives of Recipients and Providers of Peer Support: Qualitative Study
Source: JMIR Diabetes. 2026 Jan 21;11:e72779. doi: 10.2196/72779 (PMC12822868; doi:10.2196/72779)
Supplement: Multimedia Appendix 2 [file diabetes-v11-e72779-s002.docx]

**Multimedia Appendix 3**Interviewed participants’ baseline characteristics compared to non-respondents from the larger pilot participant population. Mann–Whitney U tests were applied for continuous variables and Fisher’s Exact Test for categorical variables.

|  | **Focus Group Participants**  **(*n* = 32)** | **Non-respondents**  **(*n* = 50)** | **p-Value** |
| --- | --- | --- | --- |
| **Age (years), mean ± SD** | 48 (± 16.3) | 38 (± 13.4) | *p* = 0.005 |
| **Diabetes Duration (years), mean ± SD** | 24 (± 18.1) | 20 (±12.9) | *p* = 0.61 |
| **Women, *n* (%)** | 26 (81%) | 36 (64%) | *p* = 0.43 |
| **Marital Status, *n* (%)** |  |  | *p* = 0.77 |
| Never Married | 9 (28%) | 17 (34%) |  |
| Married/living with a partner | 20 (63%) | 30 (60%) |  |
| Separated/Divorced/Widow | 3 (9%) | 3 (6%) |  |
| **Ethnicity, *n* (%)** |  |  | *p* = 0.74 |
| Aboriginal | 1 (3%) | 2 (4%) |  |
| Mixed | 1 (3%) | 6 (12%) |  |
| East Asian (Chinese, Korean, Japanese) | 1 (3%) | 2 (4%) |  |
| South Asian | - | 1 (2%) |  |
| Caucasian | 29 (91%) | 39 (78%) |  |
| **Education, *n* (%)** |  |  | *p* = 0.11 |
| High school graduate (or equivalent) | 3 (9%) | 9 (18%) |  |
| Some college or technical school | 7 (22%) | 14 (28%) |  |
| College Graduate | 10 (31%) | 20 (40%) |  |
| Graduate degree(s) | 12 (38%) | 7 (14%) |  |
| **Pre-tax Household Income, *n* (%)** |  |  | *p* = 0.72 |
| < $70,000 | 10 (31%) | 18 (36%) |  |
| > $70,000 | 17 (53%) | 22 (44%) |  |
| Decline to answer | 5 (16%) | 10 (20%) |  |
| **Employment, *n* (%)** |  |  | *p* = 0.54 |
| Full-time job | 12 (38%) | 25 (50%) |  |
| Part-time job | 6 (19%) | 7 (14%) |  |
| Retired | 6 (19%) | 6 (12%) |  |
| Other | 7 (22%) | 12 (24%) |  |
| Decline to answer | 1 (3%) | - |  |

Footnote: *p < 0.05 considered statistically significant.*
